# Design of double sampling inspection plans for life tests under time censoring based on Pareto type IV distribution

**DOI:** 10.1038/s41598-022-11834-0

**Published:** 2022-05-13

**Authors:** C. R. Saranya, R. Vijayaraghavan, K. Sathya Narayana Sharma

**Affiliations:** 1 Department of Statistics, KSMDB College, Sasthamcotta, Kerala India; 2 Department of Statistics, Bharathiar University, Coimbatore, 641 046 India; 3 Department of Mathematics, School of Advanced Sciences, Vellore Institute of Technology, Vellore, Tamil Nadu 632014 India

**Keywords:** Engineering, Materials science, Mathematics and computing

## Abstract

Sampling inspection plans for life tests, called reliability sampling plans, are generally employed to determine the acceptance or non-acceptance of the lot(s) of finished products by performing tests on the sampled items, measuring the lifetime of the items and observing the number of failures of items. Lifetime of individual items is a prime quality characteristic that can be treated as a continuous random variable and can be modeled by an appropriate probability distribution. In this article, double sampling plans for life tests under time censoring with a provision to draw two random samples and to admit a maximum of one failure in the combined samples are formulated assuming that the lifetime random variable follows a Pareto type IV distribution. A methodical procedure for the selection of the plan parameters using reliable life criterion with the desired discrimination protecting the interests of the producer and the consumer in terms of the acceptable reliable life and unacceptable reliable life is evolved. The operating ratio is used as a measure of discrimination in designing the proposed reliability sampling plans.

## Introduction

Reliability sampling plan, also termed as life test sampling plan, is a procedure that is adopted to draw a decision on the acceptability or non-acceptability of the lot(s) of the manufactured items based on the information provided by the tests on the sampled products or items. The lifetime of the product is measured from the tests on the individual items and is considered as the prime quality characteristic as well as the continuous random variable, which is described by an appropriate probability distribution. Fertig and Mann^1^ opine that a life test sampling plan is a technique for making decision on the inspected lot based on the sample(s) and employs the concept of censoring to manage the testing time at an appropriate level. In the literature of reliability sampling, four distinct censoring schemes are generally focused, viz., time censoring, failure-censoring, hybrid censoring and progressive censoring. Time censoring and failure censoring schemes are termed as time truncated and failure truncated (type I and type II) schemes. In a time terminated life test, a given sample of *n* items is tested until a pre-assigned termination time, *t*, is reached and then the test is terminated. In a failure terminated life test, a given sample size, *n*, is tested until the failure occurs and then the test is terminated. As type I and type II censoring schemes do not have the flexibility of allowing removal of units at points other than the terminal point of the experiment, when practical situations warrant the removal of surviving units at points other than the final termination, a progressive censoring scheme would be adopted as an alternative scheme. See, Balakrishnan and Aggarwala^2^. The mixture of type I and type II censoring schemes is known as hybrid censoring scheme, which is considered when cost of inspection, products and product reliabilities are high.

The basic notion and theoretical development of life test sampling plans with particular reference to exponential and Weibull distributions are found in Epstein^3,4^, Handbook H-108^5^, and Goode and Kao^6–8^. The life test sampling plans based on normal and lognormal distributions have been developed by Gupta^9^. A detailed description on the construction of life test sampling plans is provided by Schilling and Neubauer^10^. The recent literature in the studies relating to the construction of reliability sampling plans include the works of Wu and Tsai^11^, Wu et al.^12^, Kantam et al.^13^, Tsai and Wu^14^, Balakrishnan et al.^15^, Aslam et al.^16^, Kalaiselvi and Vijayaraghavan^17^, Kalaiselvi et al.^18^, Loganathan et al.^19^, Aslam et al.^20^, Hong et al.^21^, Vijayaraghavan et al.^22^, Vijayaraghavan and Uma^23,24^ and Vijayaraghavan et al.^25,26^.

Pareto distribution, introduced by Pareto^27^, is a skewed and heavy-tailed distribution. It is considered as a lifetime distribution and frequently used as a model for survival-type data. One may refer to Davis and Feldstein^28^, Wu^29^, Hossain and Zimmer^30^, Howlader and Hossain^31^, Wu and Chang^32^, Kus and Kaya^33^ and Abdel-Ghaly et al.^34^ for the details pertaining to the theory and applications of Pareto distribution. Nadarajah and Kotz^35^ considered a class of Pareto distributions and derived the corresponding forms for applications in reliability.

Pareto distribution of the first kind (type I) is the earliest form which has drawn applications in a wide range of areas. According to Arnold^36^, Pareto distribution of second kind (type II), also called Lomax distribution, is well adapted for modeling reliability problems as its properties are easily interpretable. Pareto distribution of third kind (type III) is considered as the generalized Pareto distribution. Arnold^36^ defined the Pareto distribution of fourth kind (type IV) and has observed that the Pareto distributions of the first, second and third types are the particular cases of the fourth type. Singh and Maddalla^37^ pointed out that the Pareto distribution of the fourth kind would result in decreasing failure rates. Johnson et al.^38^ observed that the Pareto distribution of the fourth kind is related to the beta distribution of the second kind, is more flexible and has wider applicability.

Due to the possibility of decreasing failure rates, the use of Pareto distribution of the fourth kind would be much helpful for practitioners to adopt in real-life phenomena and may be used as an alternative to other heavy tailed distributions. Applications of various continuous type distributions as lifetime distributions are seen in the literature of product control, particularly in reliability sampling plans. In the following subsections, double sampling plans for life tests under time censoring with a provision to draw two random samples and to admit a maximum of one failure in the combined samples are formulated assuming that the lifetime random variable follows a Pareto type IV distribution. A methodical procedure for the selection of the plan parameters using reliable life criterion with the desired discrimination protecting the interests of the producer and the consumer in terms of the acceptable reliable life and unacceptable reliable life is evolved. The operating ratio is used as a measure of discrimination in designing the proposed reliability sampling plans.

## Double sampling inspection plans for life tests

Double sampling plan (DSP) for life tests is an extension of single sampling plans and consists of a specific rule in which a second sample is drawn from the lot before it can be sentenced. It can be formulated in the following manner:

Suppose, a random sample of $n_{1}$ items is drawn from a lot and the items are placed for a life test and the experiment is stopped at a predetermined time, *T*. The number of failures occurred until the time point *T* is observed, and let it be $m_{1} .$ The lot is accepted if $m_{1}$ is equal to or less than the first acceptance number, say,$a_{1} .$ If $m_{1}$ is equal to or greater than the first rejection number $r_{1} ,$ the lot is rejected. If $a_{1} < m_{1} < r_{1} ,$ a second sample of $n_{2}$ items is taken and the number of failures,$m_{2} ,$ is observed. If the cumulative number of failures, $m_{1} + m_{2} ,$ found in the first and second samples is equal to or less than the second acceptance number,$a_{2} ,$ the lot is accepted. If $m_{1} + m_{2}$ is equal to or greater than $r_{1} ,$ the lot is rejected.

Thus, the double sampling plan for life tests is represented by the parameters $n_{1} ,\;n_{2} ,\;a_{1} ,\;r_{1}$ and $a_{2} ,$ where $n_{1}$ and $n_{2}$ are the number of items in the first and second samples, respectively, $a_{1}$ and $a_{2}$ are the allowable number of failures, called acceptance numbers, in the first sample and in the combined samples, respectively, and $r_{1} = a_{2} + 1$ is the rejection number. The plan, designated by $DSP - (n_{1} ,\;n_{2} ,\;a_{1} ,\;a_{2} ),$ is applied under the general conditions for application of sampling inspection for isolated lots.

The performance of $DSP - (n_{1} ,\;n_{2} ,\;a_{1} ,\;a_{2} )$ adopted in life testing is measured by the associated operating characteristic (OC) function, denoted by $P_{a} (p),$ which gives the probability of accepting a lot as a function of the failure probability *p*, and the average sample number function, denoted by $ASN(p),$ which yields the average number of items to be inspected under the plan for taking a decision about the lot. They are, respectively, expressed by

1 $$P_{a} (p) = F(\left. {a_{1} } \right|n_{1} ) + \sum\limits_{{m_{1} = a_{1} + 1}}^{{r_{1} - 1}} {p(\left. {m_{1} } \right|} n_{1} )F(\left. {a_{2} - m_{1} } \right|n_{2} )$$

2 $${\text{and}}\quad ASN(p) = n_{1} + n_{2} \sum\limits_{{m_{1} = a_{1} + 1}}^{{r_{1} - 1}} {p(\left. {m_{1} } \right|} n_{1} )$$

where $p(m|n)$ is the probability of observing *m* failures in a random sample of $n$ items and $F(\left. a \right|n) = \sum\nolimits_{m = 0}^{a} {p(m\,|\,n)} .$

It may be noted that under the conditions of binomial and Poisson distributions, the expressions for $p(m\,|\,n)$ are respectively given by

3 $$p(m|n) = \left( \begin{gathered} n \hfill \\ m \hfill \\ \end{gathered} \right)p^{m} (1 - p)^{n - m} ,\,\,\,{\text{for}}\,\,\,m = 0,\;1,\;2, \ldots ,\;n.$$

4 $${\text{and}}\quad p(m|n) = e^{ - np} \frac{{(np)^{m} }}{m\;!},\quad {\text{for}}\quad m = 0,\;1,\;2, \ldots .$$

Thus, the acceptance probabilities in double sampling plans under the conditions of binomial and Poisson distributions can be determined by substituting (3) and (4) in (1), respectively. In the context of life testing sampling plans, the failure probability, *p*, is defined by the proportion of product failing before time *t*, and hence, the expression for $p$ is defined by the cumulative probability distribution of *T*.

## Double sampling plans for life tests with zero or one failure

**Figure 1:**
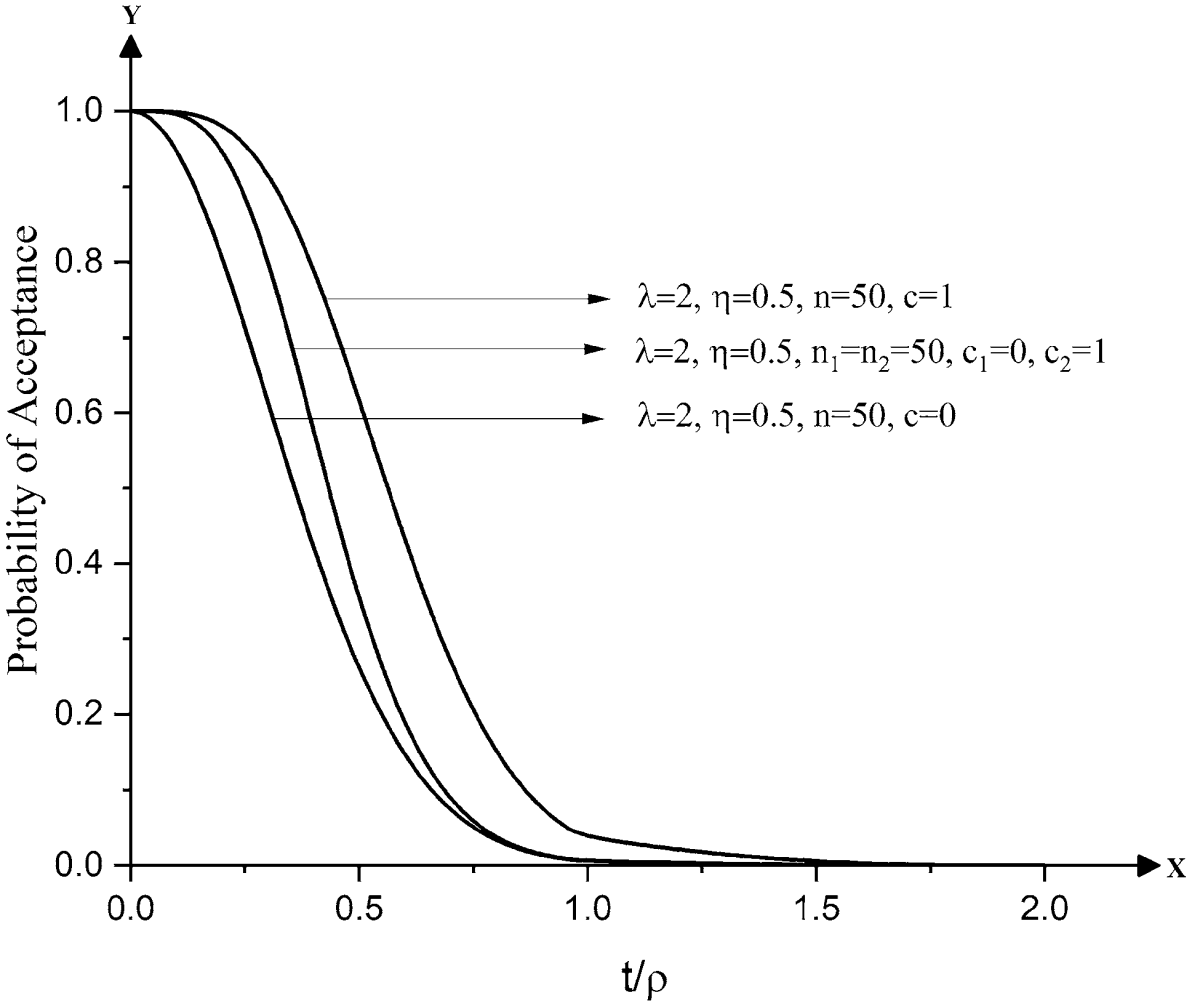
Operating characteristic curves of single and double sampling plans for life tests based on Pareto type IV distribution having smaller values of acceptance numbers.

When sampling plans for life tests are required for product characteristics that involve costly or destructive testing, and when small samples are to be involved, a sampling plan with zero or fewer failures in the samples is often employed. Dodge^39^ observed that a single sampling plan by attributes with zero acceptance number is not desirable as it seldom protects the interests of the producer. It is demonstrated in Fig. 1 that single sampling plans for life tests with zero failures or zero acceptance number, designated by $SSP - (n,\;0),$ are not desirable as they do not provide protection to the producer against the acceptable reliable life of the product. The operating characteristic curves of such sampling plans having zero failures are uniquely in poor shape, which does not ensure protection to producers, but safeguard the interests of consumers against unacceptable reliable life of the product. It can be demonstrated that single sampling plans admitting one or more failures in a sample of items lack the undesirable characteristics of $SSP - (n,\;0),$ but require larger sample sizes. This shortcoming can be overcome, to some extent, if one follows double sampling plans with a maximum of one failure in the random samples drawn from the submitted lot.

In small sample situations, single sampling plans with a fewer number of failures such as $c = 0$ and $c = 1$ can be used. But, the OC curves of $c = 0$ and $c = 1$ plans would reveal a fact that there will be a conflicting interest between the producer and the consumer as $c = 0$ plans provide protection to the consumer with lesser risk of accepting the lot having unacceptable reliable life of the product while $c = 1$ plans offer protection to the producer with lesser risk of rejecting the lot having acceptable reliable life. Such conflict can be invalidated if one is able to design a life test plan having its OC curve lying between the OC curves of $c = 0$ and $c = 1$ plans.

It can also be observed from Fig. 1 that there is a wider gap to be filled between the OC curves of $c = 0$ and $c = 1$ plans. Hence, it is, obviously, desirable to determine a plan whose OC curve is expected to lie between $c\, = \,0$ and $c\, = 1$ plans. A double sampling plan with $a_{1} = 0,\;r_{1} = 2$ and $a_{2} = 1,$ designated by $DSP - (n_{1} ,n_{2} ),$ overcomes the shortcoming of $c = 0$ plans to a greater extent by providing a desirable shape of the OC curve, which is considered as favorable to both producer and consumer. It can also be realized that the OC curves of $DSP - (n_{1} ,n_{2} )$ lie between the OC curves of $c\, = \,0$ and $c\, = 1$ plans. A special feature of $DSP - (n_{1} ,n_{2} )$ is that its OC curve coincides with the OC curve of $c\, = 1$ single sampling plan at the upper portion and coincides with the OC curve of $c\, = \,0$ single sampling plan at the lower portion. This feature would be of much help in selection of an optimum $DSP - (n_{1} ,n_{2} )$ providing protection to the producer and consumer against rejection of the lot for the specified acceptable reliable life and against acceptance of the lot for the specified unacceptable reliable life. More details about the significance and construction of sampling plans with c = 0 and c = 1 as alternative to single sampling plans with either c = 0 or with c = 1 can be had from Govindaraju^40^, Soundararajan and Vijayaraghavan^41^ and Vijayaraghavan^42^. The operating procedure of $DSP - (n_{1} ,n_{2} )$ is as follows:

A sample of $n_{1}$ items is taken from a given lot and inspected. If no failures are found, i.e., $m_{1} = 0,$ while inspecting $n_{1}$ items, then the lot is accepted; if one failure is found, i.e., $m_{1} = 1,$ a second sample of $n_{2}$ items is taken and the number of failures, $m_{2} ,$ is observed. If no failures are found, i.e., $m_{2} = 0,$ while inspecting $n_{2}$ items, then the lot is accepted; if one or more failures are found, i.e., $m_{2}$ is greater than or equal to 1, then the lot is rejected.

Associated with $DSP - (n_{1} ,n_{2} )$ are the performance measures, called OC and ASN functions, which are, respectively, expressed by

5 $$P_{a} \left( p \right) = p(0|n_{1} ,p) + p(1|n_{1} ,p)p(0|n_{2} ,p)$$

and

6 $$ASN\left( p \right) = n_{1} + n_{2} p(0|n_{1} ,p)$$

where *p* is the proportion,$p,$ of product failing before time *t*, and $p(0|n_{1} ,p),$ $p(0|n_{2} ,p)$ and $p(1|n_{1} ,p)$ are defined either from the binomial distribution or from the Poisson distribution whose probability functions are given as expressions (3) and (4).

## Pareto distribution of fourth kind

Let *T* be a random variable representing the lifetime of the components. Assume that *T* follows a Pareto distribution of fourth kind, named as Pareto Type IV distribution. The probability density function and the cumulative distribution function of *T* are, respectively, defined by

7 $$f(t;\delta ,\theta ,\eta ,\lambda ) = \frac{\lambda }{\eta \theta }\left( {1 + \left( {\frac{t - \delta }{\theta }} \right)^{{\frac{1}{\eta }}} } \right)^{ - (\lambda + 1)} \left( {\frac{t - \delta }{\theta }} \right)^{{\frac{1}{\eta }\; - \;1}} ,\;t > \delta ,\;\theta ,\eta ,\lambda > 0$$

8 $${\text{and}}\quad F\left( {t;\delta ,\theta ,\eta ,\lambda } \right) = 1 - \left( {1 + \left( {\frac{t - \delta }{\theta }} \right)^{{\frac{1}{\eta }}} } \right)^{ - \lambda } ,\;t > \delta ;\theta ,\eta ,\lambda > 0$$

where $\delta$ is the location parameter, $\theta$ is the scale parameter, $\lambda$ is the shape parameter and $\eta$ the inequality parameter. When $\delta = 0$, (7) and (8) would become

9 $$f(t;\delta ,\theta ,\eta ,\lambda ) = \frac{\lambda }{\eta \theta }\left( {1 + \left( {\frac{t}{\theta }} \right)^{{\frac{1}{\eta }}} } \right)^{ - (\lambda + 1)} \left( {\frac{t}{\theta }} \right)^{{\frac{1}{\eta }\; - \;1}} ,\;t > 0,\;\theta ,\eta ,\lambda > 0$$

10 $${\text{and}}\quad F\left( {t;\theta ,\eta ,\lambda } \right) = 1 - \left( {1 + \left( {\frac{t}{\theta }} \right)^{{\frac{1}{\eta }}} } \right)^{ - \lambda } ,\;t > 0;\theta ,\eta ,\lambda > 0$$

The mean life, reliability function and the hazard rate for a specified time *t* under the Pareto distribution are, respectively, given by

11 $$\mu = \theta \frac{\Gamma (\eta + 1)\Gamma (\lambda - \eta )}{{\Gamma (\lambda )}},$$

$$R(t) = \left( {1 + \left( {\frac{t}{\theta }} \right)^{{\frac{1}{\eta }}} } \right)^{ - \lambda }$$

and

12 $$Z\left( t \right) = \frac{\lambda }{\eta \theta }\left( {1 + \left( {\frac{t}{\theta }} \right)^{{\frac{1}{\eta }}} } \right)^{ - 1} \left( {\frac{t}{\theta }} \right)^{{\frac{1}{\eta } - 1}}$$

where $\Gamma$ is the Gamma Function.

The reliable life is the life beyond which some specified proportion of items in the lot will survive. The reliable life associated with Pareto distribution is defined and denoted by

13 $$\rho = \theta \left( {R(t)^{{ - \frac{1}{\lambda }}} - 1} \right)^{\eta }$$

The proportion,$p,$ of product failing before time *t*, is defined by the cumulative probability distribution of *T* and is expressed by

14 $$p = P(T \le t) = F(t;\theta ,\eta ,\lambda )$$

The performance of $DSP - (n_{1} ,n_{2} )$ for life tests is measured by the associated OC function, denoted by $P_{a} (p),$ which gives the probability of accepting a lot as a function of the failure probability *p*. Under the conditions for the application of binomial and Poisson models, the expressions for $P_{a} (p)$ from (1) using (3) and (4) are, respectively, given by

15 $$P_{a} \left( p \right) = (1 - p)^{{n_{1} }} + n_{1} p(1 - p)^{{n_{1} + n_{2} - 1}}$$

and

16 $$P_{a} \left( p \right) = e^{{ - n_{1} p}} + n_{1} pe^{{ - (n_{1} + n_{2} )p}}$$

Defense Department Quality Control and Reliability Technical Report TR6^43^ proposed the reliable life criterion as one of the three reliability criteria for designing reliability sampling plans when Weibull distribution is the underlying distribution for a lifetime random variable. It used the dimensionless ratio *t* / *ρ* which is related to the cumulative probability *p*, which is the proportion of product failing before time *t*. Analogous to this case of *t*/*ρ* for Weibull distribution, double sampling plans with zero or one failure indexed by the reliable life giving protection to the producer and consumer are now determined.

## Search procedure for the selection of $DSP - (n_{1} ,n_{2} )$

In reliability sampling, a specific sampling plan for life tests can be obtained by specifying the requirements that its operating characteristic (OC) curve should pass through two points, namely, $(\rho_{0} ,\alpha )$ and $(\rho_{1} ,\beta ),$ where $\rho_{0}$ and $\rho_{1}$ are the acceptable and unacceptable reliable life, associated with the risks $\alpha$ and $\beta ,$ respectively. The quantities $\rho_{0}$ and $\rho_{1}$ in reliability sampling are the counterparts of the lot quality levels in acceptance sampling, and hence, the operating ratio,$OR = \rho_{0} /\rho_{1}$, which is the ratio of acceptable reliable life to unacceptable reliable life, can be used as the measure of discrimination just similar to the operating ratio of the limiting quality level to the acceptable quality level in acceptance sampling. An optimum double sampling plan for life tests can be obtained by satisfying the following two conditions with the fixed value of producer's and consumer's risks at *α* and *β*, respectively, with minimum ASN:

17 $$P_{a} (\rho_{0} ) \ge 1 - \alpha$$

18 $${\text{and}}\quad P_{a} (\rho_{1} ) \le \beta .$$

It may be noted that the OC function is a function of *t* / *ρ*, which corresponds to the cumulative distribution, *p*, i.e., the proportion of lot failing before time *t*. Hence, for specified values of $t/\rho_{0}$ and $t/\rho_{1}$, the optimum values of $n_{1}$ and $n_{2}$ of $DSP - (n_{1} ,n_{2} )$ for the specified requirements under the conditions of Pareto type IV distribution can be determined by using the following procedure:

- *Step 1:* Specify the value of the shape parameters $(\lambda ,\eta )$ or their estimates.
- *Step 2:* Specify the proportion, *r*, of the items that will survive in the population beyond the reliability life, *ρ*.
- *Step 3:* Specify the values of $t/\rho_{0}$ and $t/\rho_{1}$, with the associated risks $\alpha = 0.05$ and $\beta = 0.10,$ respectively, so that the operating ratio is defined by $OR = \rho_{0} /\rho_{1}$ and $t/\rho_{0}$.
- *Step 4:* Using the relationship between *p* and *ρ*, from (13) and (14), obtain $p_{0}$ and $p_{1}$ corresponding to $t/\rho_{0}$ and $t/\rho_{1}$.
- *Step 5:* Search for the values of $n_{1}$ and $n_{2}$ for the specified strength $(\rho_{0} ,\;1 - \alpha )\;$ and $(\rho_{1} ,\;\beta )$ with the values of $p_{0}$ and $p_{1}$ satisfying the conditions (17) and (18), by using the expression (15) or (16).

Based on the above procedure, fixing the value of *r* as 90%, the optimum double sampling plans for life tests under the assumption of Pareto type IV distribution are obtained for a set of values of $(\lambda ,\eta )$ such as (1, 0.5), (2, 0.5), (2, 0.6) and (2, 0.7), and for various combinations of $OR = \rho_{0} /\rho_{1}$ and $t/\rho_{0}$. These plans are provided in Tables 1, 2, 3 and 4 along with the values of minimum ASN at $t/\rho_{0}$.

**Table 1:** Optimum $DSP - (n{}_{1},n_{2} )$ for life tests based on Pareto type IV distribution having shape parameters $\lambda = 1$ and $\eta = 0.5$ (*r* = 0.90). Key: $n{}_{1},n_{2} ,ASN\;at\;p_{1}$.

*OR*	$t/\rho_{0}$									
	0.03	0.04	0.05	0.06	0.07	0.08	0.09	0.1	0.11	0.12
3.6	1839,2598, 2237	1036, 1450, 1259	663, 934, 807	461, 647, 561	340, 464, 412	261, 351, 316	206, 284, 250	168, 220, 203	139, 183, 168	117, 154, 141
3.7	1713, 2945, 2139	964, 1660, 1204	618, 1048, 770	430, 718, 534	316, 534, 394	243, 393, 301	192, 316, 238	156, 251, 193	129, 211, 160	109, 170, 134
3.8	1610, 3269, 2059	907, 1800, 1155	581, 1149, 739	404, 792, 513	297, 588, 378	228, 437, 289	181, 326, 227	147, 260, 184	121, 238, 154	102, 195, 130
3.9	1522, 3540, 1985	857, 1959, 1114	549, 1247, 713	382, 837, 492	281, 613, 362	215, 503, 282	171, 342, 217	139, 266, 175	115, 224, 145	97, 182, 122
4.0	1443, 3921, 1933	813, 2059, 1071	521, 1282, 682	362, 913, 477	267, 596, 342	204, 537, 272	162, 360, 208	131, 340, 174	109, 231, 139	92, 183, 116
4.1	1373, 3866, 1836	773, 2128, 1028	495, 1399, 663	344, 1006, 465	253, 756, 344	194, 569, 263	154, 364, 198	125, 292, 161	104, 210, 130	87, 222, 114
4.2	1307, 4434, 1816	736, 2269, 997	472, 1274, 619	328, 918, 434	241, 818, 336	185, 519, 245	147, 330, 186	119, 298, 154	99, 208, 124	83, 206, 107
4.3	1247, 4197, 1710	702, 2309, 957	450, 1315, 596	313, 869, 409	230, 756, 314	177, 421, 224	140, 344, 179	114, 246, 142	94, 246, 122	79, 281, 111
4.4	1191, 4023, 1617	671, 1946, 878	430, 1202, 558	299, 826, 387	220, 603, 285	169, 413, 214	134, 305, 167	109, 231, 134	90, 213, 113	76, 164, 94
4.5	1139, 3609, 1506	641, 2194, 865	411, 1197, 533	286, 769, 365	211, 489, 262	162, 360, 199	128, 307, 160	104, 239, 129	86, 214, 109	73, 143, 88
4.6	1090, 3498, 1432	614, 1787, 789	393, 1404, 531	274, 692, 342	202, 466, 248	155, 351, 190	123, 259, 149	100, 202, 121	83, 161, 100	70, 133, 84
4.7	1044, 3480, 1372	588, 1795, 758	377, 1054, 477	262, 787, 337	193, 518, 243	148, 406, 187	117, 414, 157	95, 321, 126	79, 189, 98	67, 132, 80
4.8	1001, 3337, 1304	564, 1639, 713	361, 1338, 483	251, 1203, 361	185, 519, 233	142, 377, 177	113, 241, 136	92, 183, 109	76, 164, 92	64, 141, 78
4.9	961, 2938, 1218	541, 1690, 689	347, 956, 431	241, 818, 313	178, 426, 216	137, 292, 163	108, 277, 133	88, 194, 106	73, 156, 87	62, 112, 73
5.0	923, 2817, 1161	520, 1470, 644	333, 1025, 420	232, 601, 283	171, 409, 206	131, 340, 160	104, 239, 125	85, 164, 100	70, 161, 84	59, 136, 71
5.1	887, 2823, 1117	500, 1374, 612	320, 1063, 407	223, 584, 271	164, 462, 202	126, 320, 153	100, 231, 120	81, 215, 99	67, 261, 89	57, 116, 67
5.2	853, 2944, 1084	481, 1321, 585	308, 931, 382	215, 503, 255	158, 403, 190	121, 372, 151	96, 251, 116	78, 200, 94	65, 136, 76	55, 106, 64
5.3	821, 3114, 1057	463, 1288, 561	297, 770, 356	207, 485, 244	152, 413, 184	117, 269, 138	93, 192, 108	75, 224, 93	63, 116, 73	53, 102, 61
5.4	805, 1380, 908	446, 1256, 538	286, 769, 343	199, 528, 238	147, 330, 172	113, 241, 131	89, 257, 109	73, 143, 84	60, 162, 73	51, 102, 59
5.5	805, 952, 876	430, 1202, 516	276, 695, 326	192, 487, 227	141, 497, 177	109, 231, 126	86, 214, 102	70, 161, 82	58, 139, 69	49, 110, 57
5.6	805, 747, 861	415, 1117, 492	266, 719, 316	185, 519, 221	137, 292, 158	105, 235, 122	83, 206, 98	68, 131, 78	56, 133, 66	48, 80, 54
5.7	786, 682, 836	400, 1318, 488	257, 655, 301	179, 433, 208	132, 301, 153	101, 275, 120	80, 230, 96	65, 193, 78	54, 140, 64	46, 88, 53
5.8	742, 740, 793	387, 1031, 454	248, 682, 292	173, 410, 200	127, 380, 152	98, 216, 113	78, 154, 89	63, 151, 73	53, 92, 60	44, 122, 53
5.9	702, 810, 756	374, 1003, 437	240, 604, 278	167, 423, 194	123, 312, 143	95, 194, 108	75, 174, 86	61, 139, 70	51, 96, 58	43, 82, 49
6.0	671, 848, 725	361, 1339, 442	232, 601, 269	162, 360, 184	119, 298, 138	92, 183, 104	73, 143, 82	59, 136, 68	49, 110, 56	42, 70, 47
6.1	642, 898, 697	350, 929, 405	224, 773, 270	156, 471, 184	115, 319, 134	89, 179, 100	70, 200, 82	57, 145, 66	48, 84, 54	40, 94, 46
6.2	613, 1002, 671	339, 870, 389	217, 655, 255	151, 481, 179	112, 237, 126	86, 184, 97	68, 160, 78	56, 98, 62	46, 100, 52	39, 77, 44
6.3	590, 1061, 650	328, 918, 379	211, 489, 239	147, 330, 166	108, 277, 124	83, 206, 95	66, 147, 75	54, 103, 60	45, 81, 50	38, 68, 42
6.4	569, 1125, 630	318, 857, 364	204, 537, 233	142, 377, 163	105, 235, 118	81, 161, 90	64, 141, 72	52, 120, 59	43, 129, 51	37, 63, 41
6.5	549, 1247, 614	308, 931, 357	198, 490, 224	138, 325, 155	102, 216, 114	78, 200, 89	62, 144, 70	51, 91, 56	42, 89, 47	36, 59, 40

**Table 2:** Optimum $DSP - (n{}_{1},n_{2} )$ for life tests based on Pareto type IV distribution having shape parameters $\lambda = 2$ and $\eta = 0.5$(*r* = 0.90). Key: $n{}_{1},n_{2} ,ASN\;at\;p_{1}$.

*OR*	$t/\rho_{0}$									
	0.05	0.075	0.1	0.125	0.15	0.175	0.2	0.225	0.25	0.275
3.6	681, 950, 827	303, 423, 368	171, 235, 208	110, 147, 133	77, 99, 93	57, 71, 69	44, 53, 53	35, 41, 42	29, 30, 34	24, 26, 29
3.7	634, 1078, 790	282, 483, 352	159, 270, 199	102, 173, 128	72, 104, 88	53, 77, 65	41, 56, 50	32, 52, 40	27, 32, 32	22, 30, 27
3.8	596, 1183, 759	265, 537, 339	150, 276, 189	96, 185, 122	67, 124, 85	50, 78, 61	38, 69, 48	31, 41, 37	25, 36, 31	21, 28, 26
3.9	563, 1300, 734	251, 544, 323	142, 278, 179	91, 183, 116	64, 108, 79	47, 84, 59	36, 69, 46	29, 45, 36	24, 32, 29	20, 26, 24
4.0	534, 1387, 708	238, 570, 310	134, 338, 177	86, 213, 113	60, 138, 78	45, 73, 55	34, 79, 45	27, 61, 35	22, 49, 29	19, 25, 23
4.1	508, 1379, 674	226, 640, 303	128, 280, 162	82, 190, 105	57, 147, 75	42, 114, 56	33, 53, 40	26, 47, 32	21, 44, 27	18, 25, 22
4.2	484, 1345, 639	216, 510, 275	122, 267, 153	78, 201, 102	55, 100, 67	40, 126, 55	31, 64, 39	25, 40, 30	20, 45, 26	17, 26, 21
4.3	462, 1232, 598	206, 497, 261	116, 298, 149	75, 149, 92	52, 119, 66	39, 64, 47	30, 50, 36	24, 37, 29	19, 61, 26	16, 31, 20
4.4	441, 1239, 573	197, 452, 246	111, 261, 139	71, 216, 95	50, 97, 61	37, 68, 45	29, 42, 34	23, 34, 27	19, 25, 22	16, 19, 19
4.5	421, 2076, 632	188, 472, 237	106, 267, 134	68, 179, 87	48, 87, 58	35, 89, 45	27, 61, 34	22, 33, 26	18, 26, 21	15, 22, 18
4.6	403, 1481, 548	180, 444, 224	102, 211, 123	65, 202, 85	46, 82, 55	34, 60, 41	26, 51, 32	21, 33, 25	17, 29, 20	14, 29, 17
4.7	386, 1624, 539	172, 534, 223	97, 302, 126	63, 120, 75	44, 82, 52	33, 50, 38	25, 47, 30	20, 34, 24	16, 49, 21	14, 18, 16
4.8	371, 966, 459	165, 482, 209	93, 306, 121	60, 138, 73	42, 86, 50	31, 64, 37	24, 45, 29	19, 39, 23	16, 22, 19	13, 24, 16
4.9	356, 933, 438	159, 361, 191	90, 185, 107	58, 111, 68	40, 126, 52	30, 54, 35	23, 45, 28	19, 24, 22	15, 27, 18	13, 16, 15
5.0	342, 883, 417	152, 504, 195	86, 213, 105	56, 98, 65	39, 71, 46	29, 48, 34	22, 49, 27	18, 26, 21	15, 19, 17	12, 23, 15
5.1	328, 1142, 421	147, 320, 174	83, 177, 98	53, 159, 67	37, 104, 46	28, 44, 32	22, 29, 25	17, 31, 20	14, 23, 16	12, 15, 14
5.2	316, 865, 384	141, 346, 169	80, 163, 93	51, 154, 64	36, 69, 42	27, 42, 31	21, 30, 24	17, 21, 19	14, 17, 16	11, 30, 14
5.3	304, 895, 372	136, 308, 160	77, 159, 90	50, 85, 57	35, 58, 40	26, 41, 30	20, 32, 23	16, 25, 19	13, 21, 15	11, 16, 13
5.4	293, 819, 353	131, 300, 154	74, 165, 87	48, 87, 55	34, 51, 38	25, 40, 29	19, 39, 22	15, 46, 19	13, 16, 15	11, 12, 12
5.5	283, 694, 333	126, 323, 149	71, 216, 87	46, 95, 53	32, 74, 38	24, 41, 28	19, 25, 21	15, 22, 17	12, 23, 14	10, 19, 12
5.6	273, 670, 319	122, 267, 141	69, 144, 79	45, 73, 51	31, 64, 36	23, 45, 27	18, 29, 21	14, 39, 17	12, 16, 14	10, 14, 12
5.7	263, 744, 313	118, 245, 135	67, 124, 76	43, 83, 49	30, 60, 35	22, 61, 27	17, 43, 20	14, 20, 16	12, 13, 13	10, 11, 11
5.8	254, 727, 301	114, 236, 130	64, 168, 75	42, 68, 47	29, 57, 33	22, 32, 25	17, 24, 19	14, 16, 16	11, 17, 13	9, 20, 11
5.9	246, 603, 284	110, 239, 125	62, 145, 72	40, 85, 46	28, 58, 32	21, 35, 24	16, 33, 19	13, 21, 15	11, 13, 12	9, 13, 10
6.0	238, 570, 273	106, 267, 123	60, 138, 69	39, 71, 44	27, 61, 31	20, 45, 23	16, 22, 18	13, 16, 15	11, 11, 12	9, 10, 10
6.1	230, 589, 265	103, 220, 116	58, 140, 67	38, 63, 42	26, 94, 32	20, 28, 22	15, 31, 17	12, 24, 14	10, 16, 12	9, 9, 10
6.2	223, 527, 253	100, 199, 112	56, 168, 66	37, 57, 41	26, 38, 29	19, 33, 21	15, 21, 17	12, 17, 14	10, 12, 11	8, 15, 9
6.3	216, 510, 245	96, 290, 112	55, 100, 61	35, 89, 40	25, 40, 28	19, 24, 21	14, 39, 17	12, 13, 13	10, 10, 11	8, 11, 9
6.4	209, 534, 238	93, 306, 110	53, 109, 59	34, 79, 39	24, 45, 27	18, 29, 20	14, 21, 16	11, 21, 13	9, 18, 11	8, 9, 9
6.5	204, 409, 226	91, 183, 101	51, 154, 60	33, 75, 37	23, 64, 27	18, 22, 20	14, 17, 15	11, 15, 12	9, 13, 10	8, 8, 9

**Table 3:** Optimum $DSP - (n{}_{1},n_{2} )$ for life tests based on Pareto type IV distribution having shape parameters $\lambda = 2$ and $\eta = 0.6$ (*r* = 0.90). Key: $n{}_{1},n_{2} ,ASN\;at\;p_{1}$.

*OR*	$t/\rho_{0}$									
	0.03	0.04	0.05	0.06	0.07	0.08	0.09	0.1	0.11	0.12
4.6	603, 789, 727	374, 485, 450	258, 335, 311	191, 243, 230	148, 187, 178	119, 147, 143	98, 120, 117	82, 104, 99	70, 89, 85	61, 74, 73
4.7	574, 857, 703	356, 525, 436	246, 357, 300	182, 260, 222	141, 200, 172	113, 160, 138	93, 132, 114	78, 112, 95	67, 92, 82	58, 80, 71
4.8	549, 923, 683	340, 575, 424	235, 388, 292	174, 278, 215	135, 210, 166	108, 172, 134	89, 139, 110	75, 113, 92	64, 98, 79	56, 77, 68
4.9	527, 987, 666	327, 596, 411	226, 400, 283	167, 294, 209	129, 236, 163	104, 174, 129	85, 158, 108	72, 118, 89	61, 114, 78	53, 94, 67
5.0	507, 1068, 652	315, 618, 400	217, 443, 278	161, 299, 202	124, 259, 160	100, 183, 126	82, 160, 104	69, 131, 87	59, 110, 75	51, 99, 65
5.1	490, 1068, 631	304, 636, 388	210, 427, 267	155, 322, 198	120, 250, 154	96, 211, 124	79, 174, 103	67, 120, 84	57, 110, 72	50, 80, 61
5.2	473, 1162, 622	293, 728, 387	203, 431, 259	150, 316, 191	116, 254, 149	93, 202, 120	77, 145, 96	65, 113, 80	55, 116, 71	48, 87, 60
5.3	458, 1159, 603	284, 688, 370	196, 472, 255	145, 329, 187	112, 286, 148	90, 204, 116	74, 173, 96	63, 109, 77	53, 142, 71	46, 112, 61
5.4	444, 1119, 580	275, 710, 361	190, 459, 246	140, 408, 190	109, 237, 138	87, 228, 115	72, 150, 91	61, 108, 75	52, 96, 64	45, 85, 56
5.5	430, 1215, 574	267, 651, 344	184, 488, 242	136, 349, 178	106, 215, 132	85, 171, 106	70, 139, 87	59, 110, 73	50, 116, 64	44, 74, 53
5.6	417, 1297, 566	259, 647, 334	179, 417, 228	132, 338, 171	102, 333, 141	82, 202, 106	68, 132, 84	57, 118, 71	49, 90, 60	42, 104, 55
5.7	405, 1209, 541	251, 734, 334	174, 389, 218	128, 368, 170	100, 198, 123	80, 167, 99	66, 130, 81	55, 149, 72	47, 129, 62	41, 85, 51
5.8	394, 1027, 507	244, 668, 317	169, 382, 211	125, 273, 155	97, 200, 120	78, 150, 95	64, 133, 79	54, 103, 66	46, 94, 57	40, 76, 49
5.9	383, 990, 489	237, 697, 312	164, 397, 207	121, 319, 156	94, 213, 117	75, 245, 102	62, 144, 78	52, 133, 67	45, 81, 54	39, 71, 47
6.0	372, 1061, 483	231, 568, 291	159, 501, 212	118, 270, 147	91, 285, 121	73, 211, 96	61, 108, 73	51, 99, 62	44, 74, 52	38, 67, 46
6.1	362, 1006, 464	224, 856, 311	155, 398, 196	115, 249, 141	89, 198, 110	71, 218, 94	59, 117, 72	50, 86, 59	42, 127, 56	37, 65, 44
6.2	352, 1110, 462	218, 981, 315	151, 370, 188	112, 239, 136	87, 173, 105	70, 130, 84	57, 148, 72	48, 113, 60	41, 101, 52	36, 64, 43
6.3	343, 976, 438	213, 524, 264	147, 365, 183	109, 237, 133	84, 238, 108	68, 132, 81	56, 109, 67	47, 94, 57	40, 92, 50	35, 64, 42
6.4	334, 996, 428	207, 619, 266	143, 392, 181	106, 245, 130	82, 202, 102	66, 141, 80	54, 164, 70	46, 85, 55	39, 88, 48	34, 66, 41
6.5	326, 842, 404	202, 531, 252	140, 306, 169	103, 282, 130	80, 188, 98	64, 173, 81	53, 112, 64	45, 78, 53	38, 87, 47	33, 72, 40
6.6	318, 794, 390	197, 507, 243	136, 349, 168	101, 214, 121	78, 184, 95	63, 122, 75	52, 96, 61	44, 74, 51	37, 91, 46	32, 96, 41
6.7	310, 796, 381	192, 521, 238	133, 301, 160	98, 259, 121	76, 189, 93	61, 146, 74	51, 87, 59	43, 70, 50	36, 110, 46	32, 50, 37
6.8	302, 884, 379	187, 709, 249	130, 278, 155	96, 211, 115	74, 220, 94	60, 115, 71	49, 120, 60	42, 68, 49	36, 57, 42	31, 54, 36
6.9	295, 794, 362	183, 467, 223	126, 517, 170	94, 190, 111	73, 140, 85	58, 155, 72	48, 103, 57	41, 67, 47	35, 58, 41	30, 61, 36
7.0	288, 781, 353	179, 417, 214	124, 259, 146	91, 285, 115	71, 150, 84	57, 118, 67	47, 94, 55	40, 66, 46	34, 61, 40	30, 44, 34
7.1	281, 853, 350	175, 393, 207	121, 260, 143	89, 240, 109	69, 180, 84	56, 103, 65	46, 89, 54	39, 66, 45	33, 68, 39	29, 48, 34
7.2	275, 710, 332	171, 383, 202	118, 270, 140	87, 228, 106	68, 132, 79	54, 164, 68	45, 85, 52	38, 67, 44	32, 96, 40	28, 57, 33
7.3	269, 660, 321	167, 384, 197	115, 306, 139	85, 230, 103	66, 159, 79	53, 122, 63	44, 83, 51	37, 70, 43	32, 52, 37	28, 41, 32
7.4	263, 643, 312	163, 405, 194	113, 241, 132	83, 261, 103	65, 126, 75	52, 108, 61	43, 82, 50	36, 77, 42	31, 57, 36	27, 47, 31
7.5	257, 655, 306	159, 501, 197	110, 285, 132	82, 160, 95	63, 169, 76	51, 99, 59	42, 83, 49	35, 101, 43	30, 75, 36	26, 70, 32

**Table 4:** Optimum $DSP - (n{}_{1},n_{2} )$ for life tests based on Pareto type IV distribution having shape parameters $\lambda = 2$ and $\eta = 0.7$(*r* = 0.90). Key: $n{}_{1},n_{2} ,ASN\;at\;p_{1}$.

*OR*	$t/\rho_{0}$									
	0.03	0.04	0.05	0.06	0.07	0.08	0.09	0.1	0.11	0.12
5.6	306, 247, 350	204, 161, 233	149, 115, 170	115, 89, 131	93, 70, 106	77, 58, 88	66, 47, 75	57, 40, 65	50, 35, 57	44, 31, 50
5.7	290, 276, 337	193, 181, 224	140, 134, 163	109, 99, 126	88, 78, 102	73, 64, 85	62, 54, 72	53, 48, 62	47, 40, 55	41, 37, 48
5.8	277, 302, 327	184, 200, 217	134, 146, 158	104, 109, 123	84, 85, 99	69, 74, 82	59, 59, 69	51, 50, 60	45, 42, 53	40, 37, 47
5.9	266, 328, 319	177, 214, 212	129, 155, 154	100, 116, 119	80, 96, 96	67, 74, 79	56, 68, 67	49, 54, 58	43, 46, 51	38, 41, 45
6.0	257, 346, 311	171, 226, 206	124, 170, 151	96, 128, 116	77, 105, 94	64, 84, 78	54, 73, 66	47, 59, 57	41, 52, 50	37, 41, 44
6.1	248, 378, 305	165, 245, 202	120, 181, 148	93, 134, 114	75, 105, 91	62, 88, 76	53, 69, 64	46, 57, 55	40, 52, 49	35, 50, 43
6.2	241, 393, 299	160, 261, 199	117, 181, 144	90, 145, 112	73, 106, 89	60, 94, 74	51, 77, 63	44, 66, 54	39, 51, 47	34, 52, 42
6.3	235, 396, 292	156, 263, 194	114, 183, 141	88, 142, 109	71, 109, 87	59, 87, 72	50, 73, 61	43, 65, 53	38, 52, 46	33, 55, 42
6.4	229, 407, 287	152, 271, 191	111, 189, 138	86, 139, 106	69, 115, 86	57, 98, 71	48, 90, 61	42, 64, 52	37, 52, 45	33, 44, 40
6.5	223, 430, 282	148, 288, 188	108, 201, 136	84, 139, 104	67, 125, 85	56, 91, 69	47, 87, 60	41, 64, 50	36, 54, 44	32, 46, 39
6.6	218, 429, 276	145, 275, 183	105, 228, 136	82, 141, 102	66, 111, 82	54, 113, 70	46, 85, 58	40, 65, 49	35, 57, 43	31, 50, 38
6.7	213, 437, 271	141, 316, 183	103, 210, 131	80, 145, 100	64, 127, 81	53, 105, 67	45, 84, 57	39, 67, 49	34, 62, 43	30, 58, 38
6.8	208, 462, 268	138, 315, 179	101, 200, 128	78, 153, 98	63, 113, 78	52, 99, 65	44, 85, 56	38, 71, 48	33, 73, 43	30, 44, 36
6.9	204, 434, 260	135, 324, 177	99, 193, 124	76, 173, 99	61, 148, 80	51, 95, 64	43, 88, 55	37, 80, 48	33, 51, 40	29, 50, 36
7.0	199, 503, 262	132, 358, 177	97, 189, 121	75, 142, 93	60, 127, 76	50, 92, 62	42, 94, 54	37, 57, 45	32, 57, 40	28, 66, 37
7.1	195, 495, 256	130, 280, 165	95, 187, 119	73, 165, 94	59, 115, 74	49, 91, 61	41, 114, 56	36, 61, 44	31, 74, 41	28, 45, 34
7.2	191, 513, 253	127, 313, 165	93, 190, 117	72, 139, 89	58, 108, 72	48, 90, 60	41, 68, 50	35, 68, 44	31, 50, 38	27, 58, 35
7.3	188, 418, 238	125, 267, 157	91, 197, 115	70, 177, 92	57, 102, 70	47, 91, 58	40, 71, 49	34, 94, 46	30, 60, 38	27, 42, 33
7.4	184, 446, 236	122, 324, 160	89, 215, 115	69, 145, 87	56, 98, 68	46, 94, 58	39, 78, 49	34, 57, 41	30, 45, 36	26, 55, 33
7.5	180, 551, 243	120, 278, 152	88, 169, 108	68, 129, 83	55, 95, 67	45, 100, 57	38, 97, 50	33, 66, 41	29, 54, 36	26, 41, 31
7.6	177, 448, 228	118, 255, 147	86, 183, 107	66, 194, 88	54, 92, 65	44, 117, 58	38, 63, 46	33, 50, 39	29, 42, 34	25, 56, 32
7.7	174, 408, 220	116, 240, 143	84, 220, 109	65, 153, 83	53, 91, 64	44, 73, 53	37, 69, 45	32, 57, 39	28, 50, 34	25, 40, 30
7.8	171, 386, 214	113, 383, 155	83, 170, 102	64, 137, 80	52, 90, 63	43, 77, 52	36, 87, 46	31, 82, 41	28, 40, 33	24, 69, 32
7.9	168, 373, 209	111, 354, 149	81, 212, 104	63, 127, 77	51, 91, 61	42, 83, 52	36, 59, 43	31, 52, 37	27, 49, 33	24, 41, 29
8.0	165, 368, 204	109, 377, 149	80, 168, 98	62, 120, 75	50, 92, 60	41, 99, 52	35, 68, 43	30, 69, 38	27, 39, 32	24, 34, 28
8.1	162, 370, 201	108, 221, 132	78, 303, 110	61, 115, 74	49, 95, 60	41, 68, 49	35, 53, 41	30, 49, 36	26, 50, 32	23, 45, 28
8.2	159, 383, 199	106, 224, 130	77, 180, 96	60, 112, 72	48, 100, 59	40, 74, 48	34, 60, 41	29, 65, 36	26, 39, 31	23, 35, 27
8.3	156, 419, 199	104, 233, 128	76, 156, 92	59, 109, 71	47, 112, 59	39, 88, 48	33, 79, 42	29, 47, 34	25, 54, 31	22, 66, 29
8.4	154, 339, 188	102, 253, 128	75, 142, 90	58, 108, 69	47, 78, 55	39, 63, 46	33, 55, 39	28, 66, 35	25, 40, 30	22, 39, 27
8.5	151, 373, 188	100, 352, 135	73, 194, 93	57, 107, 68	46, 82, 55	38, 71, 46	32, 70, 39	28, 47, 33	24, 116, 36	22, 32, 26

## Procedure for the selection of* DSP*−(n_1_, n_2_) using the tables

The parameters of a double sampling plan for life tests when the lifetime random variables follows a Pareto type IV distribution are chosen from the given tables by the following method:

- *Step 1:* Specify the values of $\lambda$ and $\eta$ or their estimates based on a past history.
- *Step 2:* Specify the test termination time, t, and the requirements $(\rho_{0} ,\;1 - \alpha )\;$ and $(\rho_{1} ,\;\beta )$.
- *Step 3:* Compute $t/\rho_{0}$ and $t/\rho_{1}$ with $\alpha = 0.05$ and $\beta = 0.10,$ respectively.
- *Step 4:* Find the operating ratio, $OR = \rho_{0} /\rho_{1}$*.*
- *Step 5:* Enter the appropriate table (among Tables 1, 2, 3 and 4) corresponding to the given set of values of $\lambda$ and $\eta ;$ choose the values of $n_{1}$ and $n_{2}$ corresponding to the value of $t/\rho_{0}$ and the operating ratio which is just closer to *OR* found in *Step 3*.

Thus, the values of $n_{1}$ and $n_{2}$ will constitute the required optimum $DSP - (n_{1} ,n_{2} )$ for life tests satisfying the given requirements. The optimum plan would admit a maximum of one failure in an accepted lot.

### Numerical illustration 1

A double sampling plan for life tests is to be instituted when the lifetime of the component is considered as a random variable which follows a Pareto type IV distribution whose shape parameters are specified as $\lambda = 1$ and $\eta = 0.5$. Assume that nearly 90% of items in the population will survive beyond the reliability life *ρ*, i.e., *r* = 0.90. It is expected that interests of the producer and the consumer are to be protected when the acceptable reliable life and the unacceptable reliable life are specified $\rho_{0} = 2500$ hours and $\rho_{1} = 500$ hours, respectively, with the associated producer’s risk of 5% and consumer’s risk of 10%.

It is desired that the life test is to be terminated at t = 200 h. From the given set of values, one finds *OR* = 5 and $t/\rho_{0} = 0.08$. Thus, entering Table 1 with $OR = \rho_{0} /\rho_{1} = 5$ and $t/\rho_{0} = 0.08$, the optimum double sampling plan is chosen having its sample sizes $n_{1} = 131$ and $n_{2} = 340,$ which yield $ASN = 160.$ One may obtain acceptable and unacceptable quality levels corresponding to $\rho_{0} = 2500$ hours and $\rho_{1} = 500$ hours using the relationship between t / ρ and *p* as 0.000711 and 0.017467, respectively. Thus, the desired plan for the given conditions is implemented as given below:

1. Choose $n_{1} = 131$ items from a lot.
2. Conduct the life test experiment on each sampled item.
3. Count the number of failures, *x*, before attaining the termination time.
4. Terminate the life test at time $t = 200$ hours.
5. If no failures are observed in the 131 items tested or until time *t* is reached, accept the lot; if there are 2 or more failures, reject the lot; if one failure is observed, select a random sample of $n_{2} = 340$ items.
6. Conduct the life test on each of the 340 items. Accept the lot, when there are no failures in the 340 items; if one or more failures are observed, reject the lot.
7. Treat the items which survive beyond time $t = 200$ hours as passed.

### Numerical illustration 2

Consider a situation in which the lifetime of an item follows the Pareto type IV distribution which has the shape parameters $\lambda$ and $\eta .$ Assume that the estimated values of $\lambda$ and $\eta$ are 2 and 0.5, respectively. The life test will be terminated at *t* = 500 h. The acceptable and unacceptable proportion of failures are prescribed as $p_{0} = 0.168\%$ and $p_{1} = 2.65\%$ with the associated producer’s and consumer’s risks specified as $\alpha = 0.05$ and $\beta = 0.10$. Corresponding to $p_{0} = 0.168\%$ and $p_{1} = 2.65\%$, one obtains $t/\rho_{0} = 0.125$ and $t/\rho_{1} = 0.5$. Hence, the desired operating ratio is obtained as $OR = \rho_{0} /\rho_{1} = 4.$ As $\lambda = 2$ and $\eta = 0.5,$ entering Table 2, the optimum double sampling plan is identified with its samples sizes given as $n_{1} = 86$ and $n_{2} = 213$ yielding ASN = 113 at $t/\rho_{0} = 0.125$. The desired sampling plan satisfies the conditions the conditions (17) and (18). The acceptable and unacceptable reliable life, corresponding to $p_{0} = 0.168\%$ and $p_{1} = 2.65\%$ are determined, $\rho_{0} = {t \mathord{\left/ {\vphantom {t {0.125}}} \right. \kern-\nulldelimiterspace} {0.125}} = 4000$ h and $\rho_{1} = {t \mathord{\left/ {\vphantom {t {0.5 = }}} \right. \kern-\nulldelimiterspace} {0.5 = }}1000$ h, respectively.

**Figure 2:**
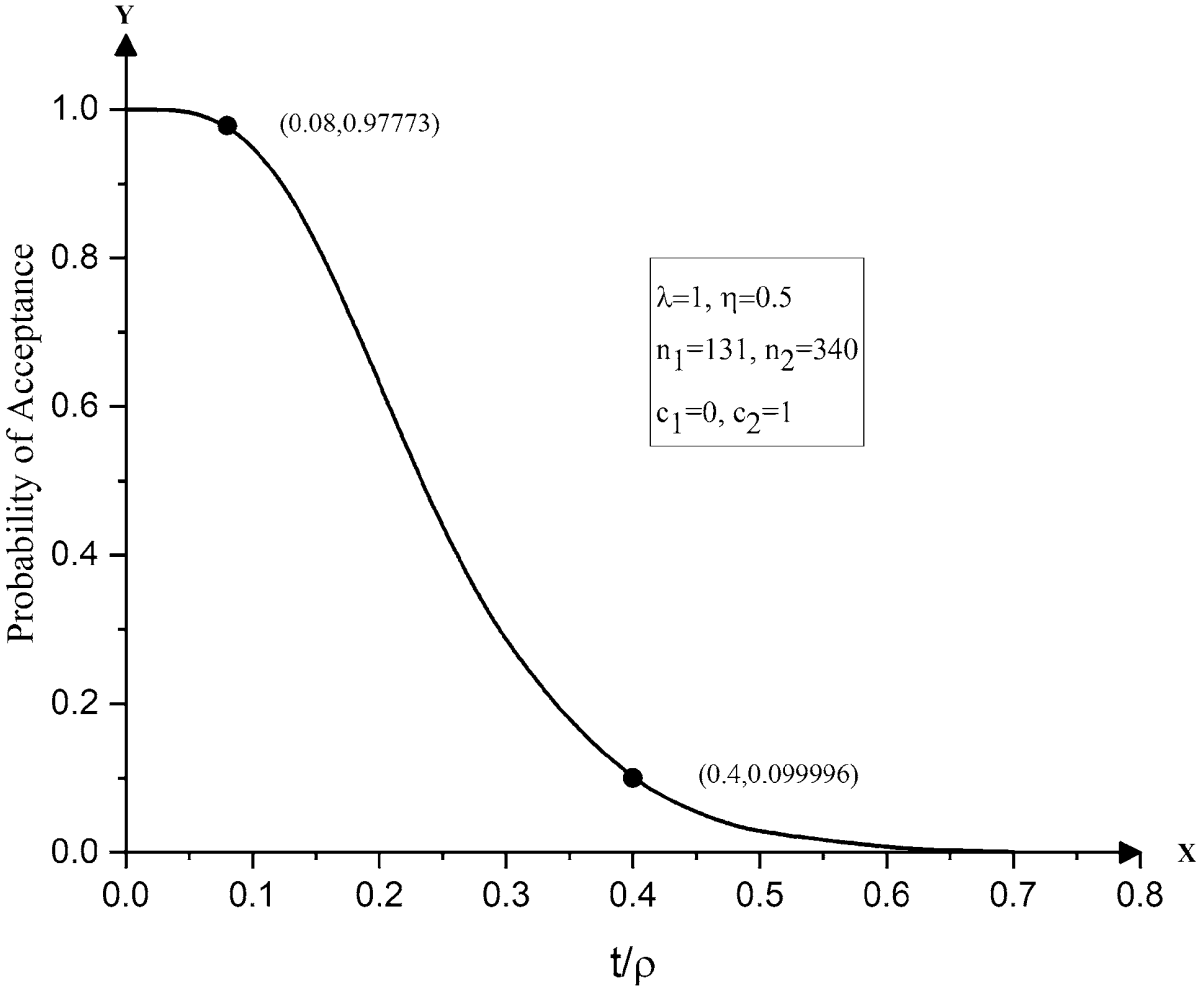
OC curves of double sampling plans for life tests based on Pareto type IV distribution with $n_{1} = 131,n_{2} = 340,\lambda = 1$ and $\eta = 0.5$.

**Figure 3:**
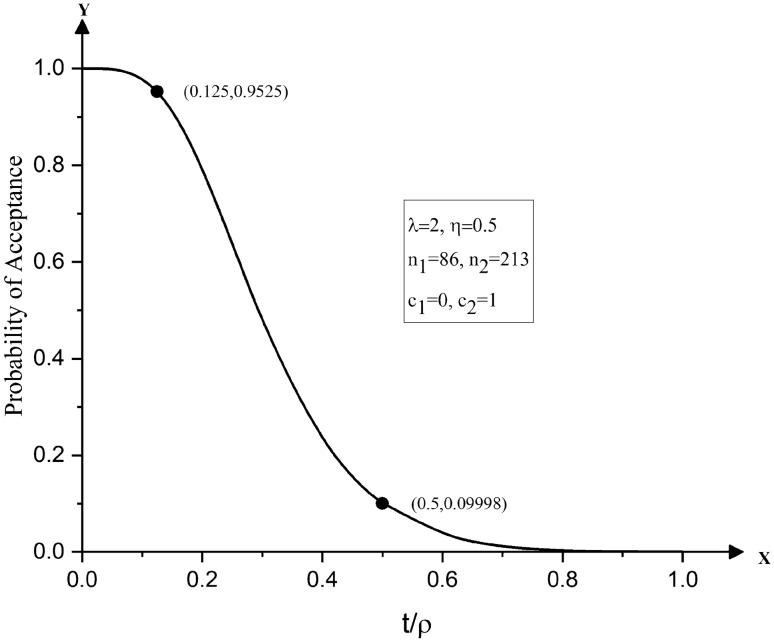
OC curves of double sampling plans for life tests based on Pareto type IV distribution with $n_{1} = 86,n_{2} = 213,\lambda = 2$ and $\eta = 0.5$.

Figures 2 and 3 display the OC curves of the double sampling plans obtained in Numerical Illustrations 1 and 2. It can be observed in Fig. 2 that the OC curve of the double sampling plan $(n_{1} = 131,n_{2} = 340)$ for life tests based on the Pareto type IV distribution passes through the desired points, namely, (0.08, 0.9777) and (0.4, 0.0999). Similarly, from Fig. 3, it can be noted that the optimum plan $(n_{1} = 86,n_{2} = 213)$ passes through the points (0.125. 0.9525) and (0.5, 0.09998).

### Numerical illustration 3

A manufacturing industry produces various models of rotating wheels which can be used for different applications. The quality levels of rotating wheels are specified in terms of the useful life which is measured in terms of the expected number of revolutions per minute. For a particular make of rotating wheel, the producer specifies that, nearly 90% or more of the items would survive beyond 1000 revolutions per minute and expects that the lot should have the probability of acceptance at 0.95, i.e., $(\rho_{0} = 1000,\alpha = 0.05)$. The consumer’s specification is that 90% or more of the items will survive 118 or lesser revolutions per minute, i.e., and 10% risk of accepting such a lot, $(\rho_{1} = 118,\;\beta = 0.10).$

From the history, it was ascertained that the quality variable of rotating wheel follows a Pareto type IV distribution with parameters $\lambda$ and $\eta$, specified 2 and 0.7. For the given situation, it is desired to institute a reliability double sampling plan. It is assumed that the life test is to be performed on rotating wheels until reaching $t = 100$ revolutions per minute (rpm) at its axis. The tested rotating wheel when it does not reach 100 revolutions per minute can be treated as a failure of the item. From the given requirements the values of $t/\rho_{0}$ and $t/\rho_{1}$ are obtained as $t/\rho_{0} = 0.1$ and $t/\rho_{1} = 0.85.$ The operating ratio is found as $OR = \rho_{0} /\rho_{1} = 8.5$. As $\lambda = 2$ and $\eta = 0.7,$ by entering Table 4 with $OR = \rho_{0} /\rho_{1} = 8.5$ and $t/\rho_{0} = 0.1$, the optimum parameters of the double sampling plan are chosen as $n_{1} = 28$ and $n_{2} = 47,$ with the associated ASN = 33 at $t/\rho_{0} = 0.1$. Thus, the desired plan is implemented is as follows:

Select a first random sample of $n_{1} = 28$ rotating wheels and conduct the life test on each of the selected item; if no failures are observed until reaching *t* = 100 revolutions per minute, accept the lot; if one failure is observed, select a second random sample of $n_{2} = 47$ items. Conduct the life test on each of the 47 items. Accept the lot, when there are no failures in the 47 items; if one or more failures are observed, reject the lot.

## Simulation study

A simulation study is carried out for comparing the results arrived in the above illustration. The simulated results are based on 10,000 runs using R programming. Initially, first random sample of size $n_{1} = 28$ is simulated from Pareto type IV distributions with the shape parameters $\lambda$ and $\eta$ are specified 2 and 0.7. The resulted simulated data are arranged in an ascending order as given below: 98.79, 100.25, 101.24, 102.25, 103.11, 104.30, 106.13, 111.38, 111.52, 113.28, 115.61, 124.66, 127.65, 130.24, 132.01, 135.11, 137.00, 139.22, 140.31, 140.54, 141.32, 146.71, 153.96, 155.49, 159.07, 180.52, 254.41, 275.76.

It can be observed that there is one failure before truncation of $t = 100$ revolutions, hence, a second random sample of $n_{2} = 47$ observations is generated from the distribution having the parameters $\lambda$ and $\eta$ specified as 2 and 0.7, respectively. The simulated data are given below in the ascending order:

101.09, 102.20, 104.79, 106.60, 107.80, 108.79, 109.05, 109.08, 109.64, 111.00, 111.30, 113.23, 113.86, 115.32, 116.56, 117.21, 117.62, 117.99, 121.55, 121.90, 122.47, 125.69, 126.87, 126.97,127.27, 131.32, 135.52, 138.34, 139.34, 142.19, 142.47, 142.89, 143.32, 143.34, 147.79, 149.53, 154.73, 155.55, 159.25, 166.36, 170.70, 180.66, 205.85, 207.36, 242.14, 269.06, 310.49.

It can be noted that the entities in the simulated data exhibit the more than *t* = 100 and no failure is observed in the second sample. Hence, the lot is treated as accepted.

## Conclusion

Double sampling plans for life tests are proposed when the lifetime random variable follows a Pareto type IV distribution. A procedure for designing the sampling plans indexed by acceptable and unacceptable reliable life for a situation involving time truncation is discussed with illustrations. Tables yielding optimum double sampling plans for life tests for a selected set of parametric values of Pareto type IV distribution. A simulation study has been carried out to demonstrate the application of the proposed plans for the industrial needs.
